# Author Correction: Meiotic protein SYCP2 confers resistance to DNA-damaging agents through R-loop-mediated DNA repair

**DOI:** 10.1038/s41467-024-48241-0

**Published:** 2024-05-07

**Authors:** Yumin Wang, Boya Gao, Luyuan Zhang, Xudong Wang, Xiaolan Zhu, Haibo Yang, Fengqi Zhang, Xueping Zhu, Badi Zhou, Sean Yao, Aiko Nagayama, Sanghoon Lee, Jian Ouyang, Siang-Boon Koh, Eric L. Eisenhauer, Dominique Zarrella, Kate Lu, Bo R. Rueda, Lee Zou, Xiaofeng A. Su, Oladapo Yeku, Leif W. Ellisen, Xiao-Song Wang, Li Lan

**Affiliations:** 1 Massachusetts General Hospital Cancer Center, Harvard Medical School, 13th Street, Charlestown, MA 02129 USA; 2 Department of Molecular Biology and Microbiology, Duke University School of Medicine, 213 Research Drive, Durham, NC 27710 USA; 3 Emory University School of Medicine, Atlanta, GA 30322 USA; 4 Ludwig Center at Harvard, Boston, MA 02215 USA; 5 UPMC Hillman Cancer Center, University of Pittsburgh, 5117 Centre Ave, Pittsburgh, PA 15232 USA; 6 Department of Pathology, University of Pittsburgh, Pittsburgh, PA 15232 USA; 7 Department of Biomedical Informatics, University of Pittsburgh, Pittsburgh, PA 15232 USA; 8 School of Cellular & Molecular Medicine, University of Bristol; University Walk, Bristol, BS8 1TD UK; 9 Division of Gynecologic Oncology, Department of Obstetrics and Gynecology, 55 Fruit St, Massachusetts General Hospital, Boston, MA 02114 USA; 10 Obstetrics, Gynecology and Reproductive Biology, Harvard Medical School, Boston, MA 02115 USA; 11 Vincent Center for Reproductive Biology, Department of Obstetrics and Gynecology, 55 Fruit St, Massachusetts General Hospital, Boston, MA 02114 USA; 12 David H. Koch Institute for Integrative Cancer Research, Department of Biology, Massachusetts Institute of Technology, Cambridge, MA 02139 USA; 13 Department of Pathology, Massachusetts General Hospital, Harvard Medical School, 55 Fruit St, Boston, MA 02114 USA; 14 Department of Pharmacology & Cancer Biology, Duke University School of Medicine, 213 Research Drive, Durham, NC 27710 USA; 15 Division of Hematology-Oncology, Massachusetts General Hospital, 55 Fruit St, Boston, MA 02114 USA; 16 Department of Medicine, Massachusetts General Hospital, 55 Fruit St, Boston, MA 02114 USA

**Keywords:** Breast cancer, DNA damage response

Correction to: *Nature Communications* 10.1038/s41467-024-45693-2, published online 21 February 2024

In the original version of the published article, the Acknowledgements section was missing a funding source number for Jian Ouyang: National Institutes of Health grant P50 CA274158 (JO).

The correct Acknowledgements paragraph is:

National Institutes of Health grant R01 GM118833 and CA282939 (LL). National Institutes of Health grant R01 CA181368, CA183976, and 1R21CA237964 (Xi. W). National Institutes of Health grant R01 GM076388 and CA197779 (L.Zo). National Institutes of Health grant P50 CA274158 (JO). Nile Albright Research Foundation and Vincent Memorial Hospital Foundation (B.R.R.).

This has now been corrected both in the HTML and the PDF versions of the article.

