# Supplementary material for: Meiotic protein SYCP2 confers resistance to DNA-damaging agents through R-loop-mediated DNA repair
Source: Nat Commun. 2024 Feb 21;15:1568. doi: 10.1038/s41467-024-45693-2 (PMC10881575; doi:10.1038/s41467-024-45693-2)
Supplement: Supplementary file 1 — Supplementary Information [file 41467_2024_45693_MOESM1_ESM.pdf]

Meiotic Protein SYCP2 Confers Resistance to DNA-Damaging Agents through R-Loop-Mediated DNA Repair

Yumin Wang[1]*, Boya Gao[1, 2]*, Luyuan Zhang[3], Xudong Wang[1], Xiaolan Zhu[1], Haibo Yang[1], Fengqi Zhang[1,2], Xueping Zhu[1], Badi Zhou[1], Sean Yao[1], Aiko Nagayama[1,4], Sanghoon Lee[5,6,7], Jian Ouyang[1], Siang-Boon Koh[8], Eric L. Eisenhauer[9,10], Dominique Zarrella[11], Kate Lu[12], Bo R. Rueda[9,10,11], Lee Zou[1,13,14], Xiaofeng A. Su[12], Oladapo Yeku[1,15,16], Leif W. Ellisen[1,4], Xiao-Song Wang[5,6,7], Li Lan[1,2†]

[1]Massachusetts General Hospital Cancer Center, Harvard Medical School; 13th Street, Charlestown, MA, 02129, USA.
[2] Department of Molecular biology and Microbiology, Duke University School of Medicine; 213 Research Drive, Durham, NC, 27710, USA
[3]Emory University School of Medicine; Atlanta, GA, 30322, USA.
[4]Ludwig Center at Harvard; Boston, MA 02215, USA
[5]UPMC Hillman Cancer Center, University of Pittsburgh; 5117 Centre Ave, Pittsburgh, PA, 15232, USA.
[6]Department of Pathology, University of Pittsburgh; Pittsburgh, PA, 15232, USA.
[7]Department of Biomedical Informatics, University of Pittsburgh; Pittsburgh, PA, 15232, USA.
[8]School of Cellular & Molecular Medicine, University of Bristol; University Walk, Bristol BS8 1TD, UK
[9]Division of Gynecologic Oncology, Department of Obstetrics and Gynecology, 55 Fruit St, Massachusetts General Hospital; Boston, MA 02114, USA
[10]Obstetrics, Gynecology and Reproductive Biology, Harvard Medical School; Boston, MA 02115, USA
[11]Vincent Center for Reproductive Biology, Department of Obstetrics and Gynecology, 55 Fruit St, Massachusetts General Hospital; Boston, MA 02114, USA
[12]David H. Koch Institute for Integrative Cancer Research, Department of Biology, Massachusetts Institute of Technology, Cambridge, MA, 02139, USA
[13]Department of Pathology, Massachusetts General Hospital, Harvard Medical School; 55 Fruit St, Boston, MA 02114, USA.
[14]Department of Pharmacology & Cancer Biology, Duke University School of Medicine, 213 Research Drive, Durham, NC, 27710, USA.
[15]Division of Hematology-Oncology, Massachusetts General Hospital; 55 Fruit St, Boston, MA, 02114, USA.
[16]Department of Medicine, Massachusetts General Hospital; 55 Fruit St, Boston, MA, 02114, USA.

* These authors contributed equally to this work.
† To whom correspondence should be addressed: li.lan@duke.edu

Supplementary Figure 1

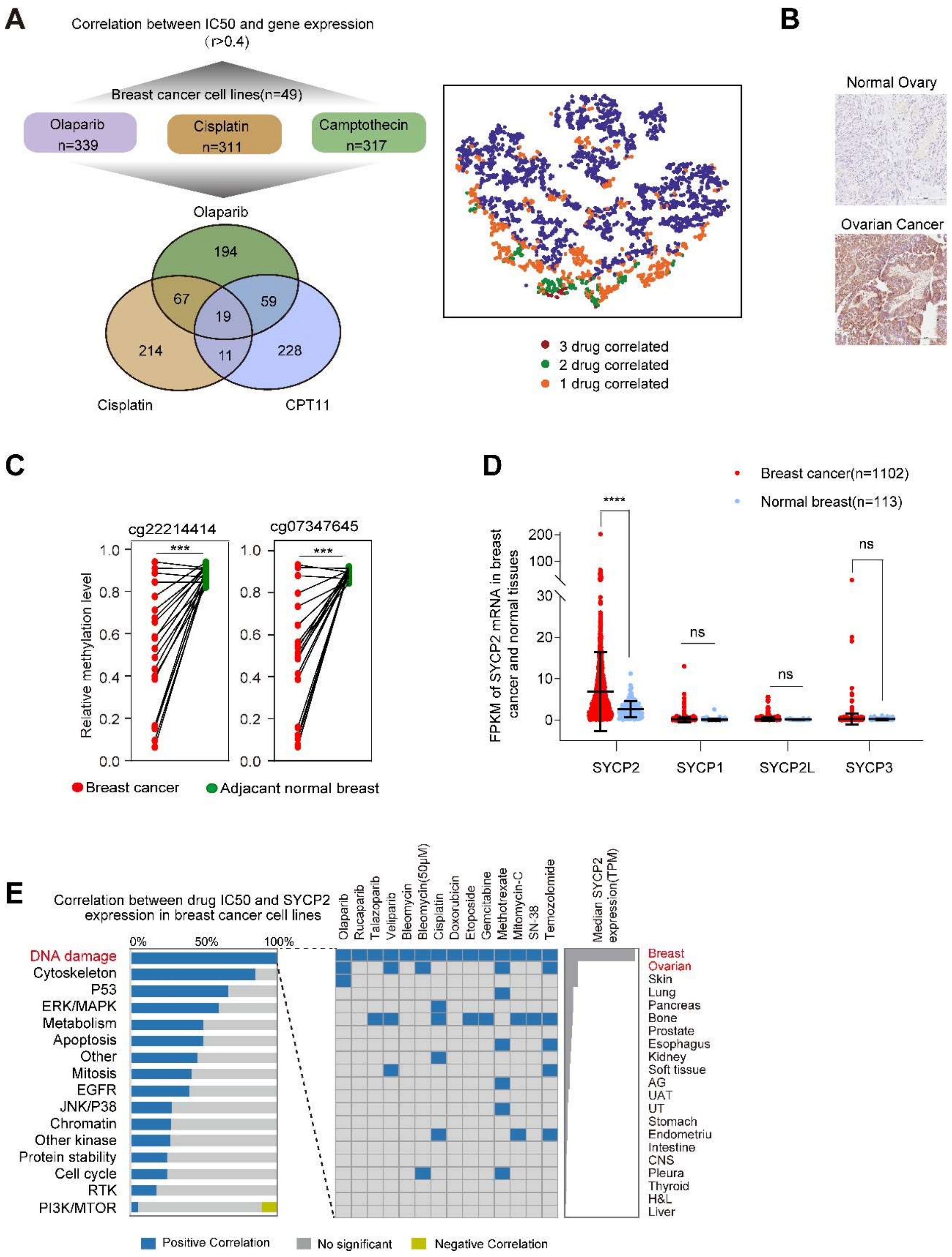

Supplementary Figure 1. SYCP2 expression in cancer associates with resistance to drugs targeting DDR pathways. A. Pearson correlation analysis using IC50 of three DDR-targeting drugs (Olaparib, Cisplatin, and CPT) and RNA seq of breast cancer cell line (n=49) from GDSC and CCLE, respectively. Cluster was evaluated

by PCA. Dimensionally reduced plot is shown the correlated genes in which Pearson correlation coefficients value >0.3, p-value <0.05 from each indicated drug group. Venn plot of correlated genes from each indicated drug-treated group are shown on the right. The plot showed one hundred fifty-two common hits. **B.** IHC of SYCP2 staining in ovarian cancer and normal ovary tissues. **C.** The relative DNA methylation levels at two randomly selected loci shown from Fig. 1E of breast tumors compared to the adjacent normal breast tissue. **D.** Comparison of SYCP2, SYCP1, SYCP2L and SYCP3 RNA expression (CCLE database) between breast cancer and normal breast samples. The analysis was normalized to Fragments Per Kilobase Million (FPKM). **E.** Pearson correlation between expression of SYCP2 and IC50 of drugs (total 251) targeting different pathways from GDSC database in breast cancer cell lines. RNA seq data of cell lines were from CCLE. Patients' expression data were from TCGA. p values in the correlation study were determined by using Pearson correlation analysis. Statistical analysis was done with the Student-t-test, ***$P < 0.001$.

Supplementary Figure 2

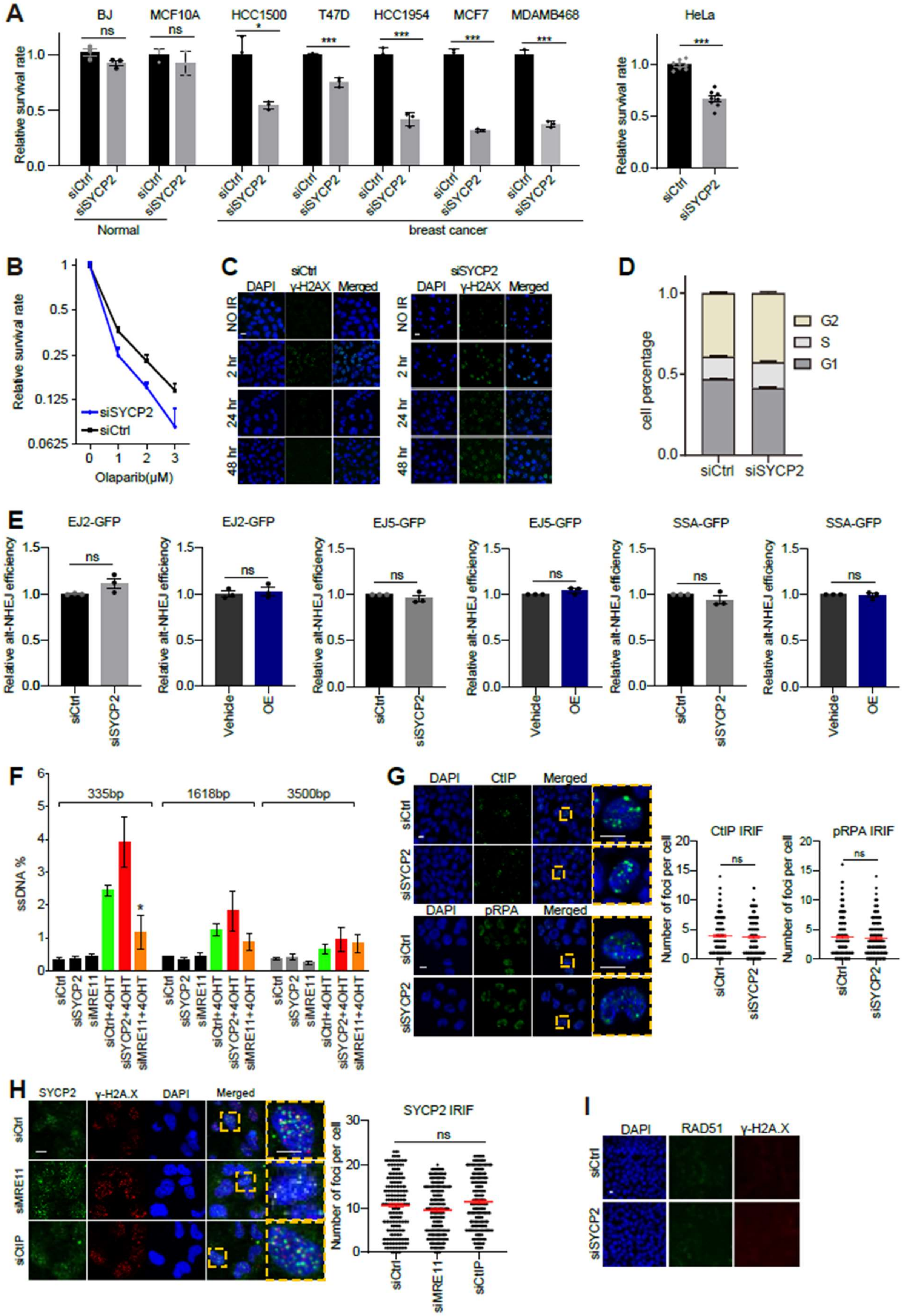

Supplementary Figure 2. SYCP2 is required for HR without affecting end resection or cell cycle progression. **A.** Cell survival rate of BJ, normal MCF10A breast cell line, breast cancer cell lines: HCC1500, T47D, HCC1954, MCF7, and MDAMB468, and HeLa cells with the treatment of siCtrl or siSYCP2 (n=3, mean +/- S.D.). **B.** Survival rate of U2OS cells with siCtrl or siSYCP2 with Olaparib at the indicated dose via colony-forming assay (n=3, mean +/- S.D.). **C.** Representative images of γH2AX IRIF in siCtrl and siSYCP2 treated U2OS cells 1 hr after 2 Gy IR. **D**. The histogram of Cell cycle distribution by propidium iodide (PI) staining and FACS analysis of U2OS SCE cells in siCtrl and siSYCP2 treated U2OS cells. **E.** Relative NHEJ frequency, alt-NHEJ frequency and SSA frequency in siCtrl or siSYCP2 (left) or empty vector or SYCP2 OE (right) using the EJ5, EJ2 and SSA reporter assay, respectively (n=3, mean +/- S.D.). **F.** End resection rate of ER-*Asi*SI U2OS cells pretreated with siSYCP2, siMRE11 or siCtrl and with or without 300 nM 4-OHT for 4 hours. Quantification of percentage of ssDNA was measured by qPCR. **G.** The numbers of IRIF of CtIP (upper) or pRPA (lower) in siCtrl and siSYCP2 treated U2OS cells 1 hr after 2 Gy IR were quantified (n=200, +/- SD). The representative images were shown on the left. **H.** The numbers of SYCP2 IRIF in siCtrl, siMRE11 or siCtIP treated U2OS cells 1 hr after 2 Gy IR were quantified (n=200, +/- SD). The representative images were shown on the left. **I.** RAD51 and γH2AX staining in siCtrl or siSYCP2 treated U2OS cells. Statistical analysis was done with the Student-t-test, $*P < 0.05$, $***P < 0.001$. Scale bar = 10 μm.

Supplementary Figure 3

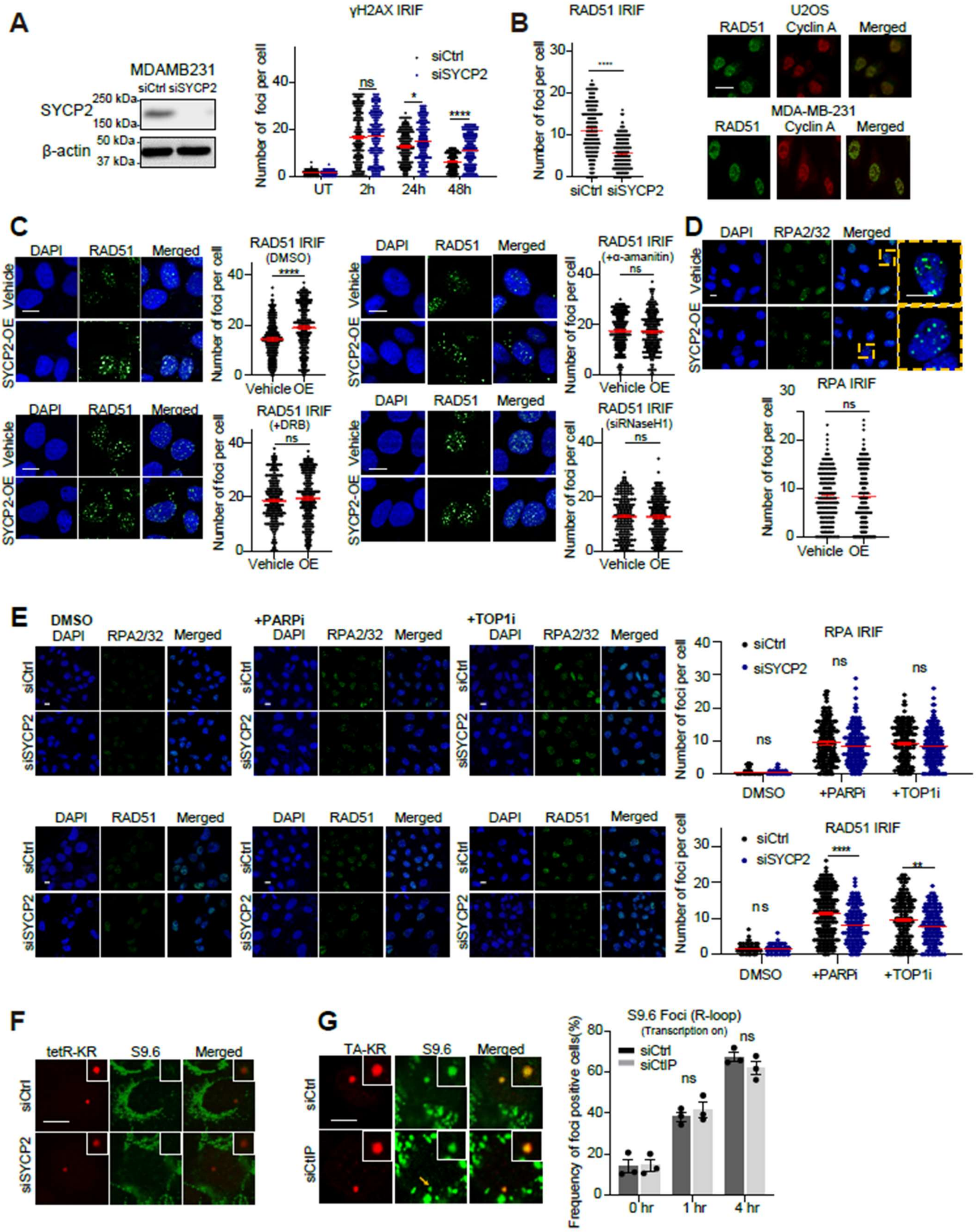

Supplementary Figure 3. SYCP2 is required for RAD51 foci formation.

A. The numbers of γH2AX IRIF in siCtrl and siSYCP2 treated MDAMB231 cells 1 hr after 2 Gy IR (n=200, +/- SD). WB of SYCP2 KD in MDAMB231 was shown. **B.** Left: The numbers of RAD51 IRIF in siCtrl and siSYCP2 treated MDAMB231 cells 1 hr after 2 Gy IR (n=200, +/- SD). Right: Representative images of Cyclin A and RAD51 in U2OS cells (left) or MDA-MB-231 cells (right) 1 hr after 2 Gy IR. **C.** The numbers of RAD51 IRIF in vehicle and SYCP2-OE U2OS cells with treatment of DMSO or 20 μM DRB or 100 μg/ml α-amanitin or siRNaseH1 1 hr after 2 Gy IR were quantified (n=200, +/- SD). Representative images of RAD51 in siCtrl and siSYCP2 U2OS cells with indicated treatment were shown on the left. **D.** The numbers of SYCP2 IRIF in siCtrl, siMRE11 and siCtIP treated U2OS cells 1 hr after 2 Gy IR were quantified (n=200, +/- SD). The representative images of SYCP2 and γH2AX IRIF in siCtrl, siMRE11 and siCtIP treated U2OS were shown on the left. **E.** The numbers of RAD51 (upper) and RPA (lower) foci in siCtrl and siSYCP2 treated U2OS cells with either 1μM PARPi or 500 nM CPT11 treatment (n=200, +/- SD). The representative images are shown on the left. **F.** Representative images of S9.6 staining in siSYCP2#2 pretreated or siCtrl U2OS-TRE cells at tetR-KR sites. **G.** U2OS-TRE cells transfected with TA-KR and siCtrl/siCtIP with or without light-activation were recovered at 1 hr and 4 hr, then fixed and stained with anti-S9.6. Frequency of S9.6 foci positive cells at TA-KR was counted. Representative images of S9.6 staining in siCtIP or siCtrl pretreated U2OS-TRE cells were shown on the left (n=3, mean+/- SD). Statistical analysis was done with the Student-t-test, $^*P < 0.05$, $^{***}P < 0.001$, **** p<0.0001. Scale bar = 10 μm.

Supplementary Figure 4

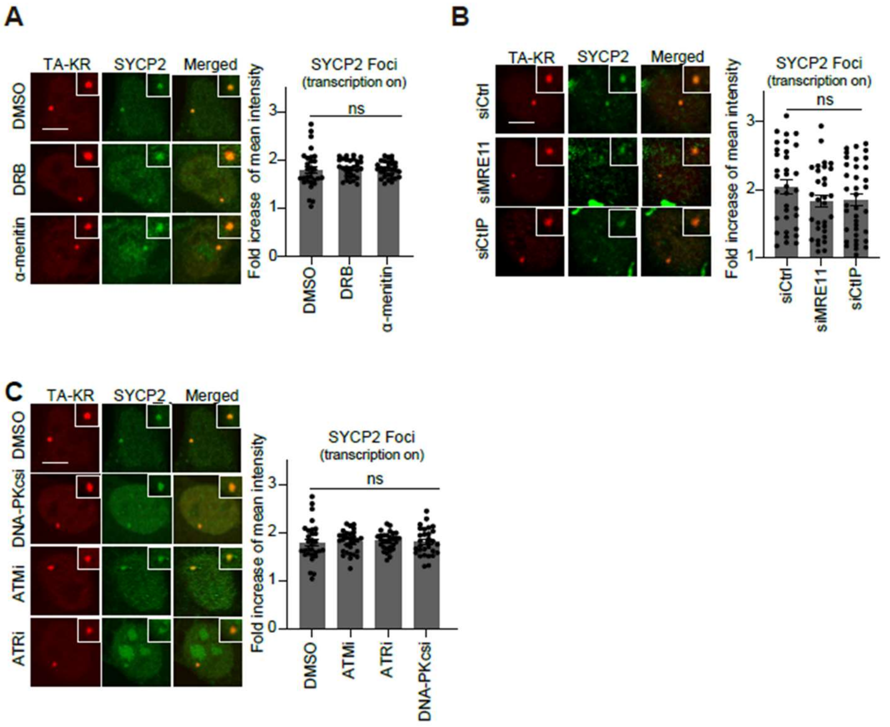

Supplementary Figure 4. Recruitment of SYCP2 is not dependent on DDR kinases or DNA ends rection. U2OS-TRE cells transfected with TA-KR and GFP-SYCP2 and treated with 20 μM DRB or 2 μM α-amanitin or DMSO **(A);** siMRE11/ siCtIP or siCtrl **(B);** and 10 μM ATMi KU55933, 10 μM ATRi CGK733, 1 μM DNA PKi KU7441 **(C)** were light-activated, recovered for 30 min, fixed. Fold increase of SYCP2 foci at sites of KR compared to background was quantified (n=30, mean+/- SD). Statistical analysis was done with the Student-t-test. Scale bar = 10 μm.

Supplementary Figure 5

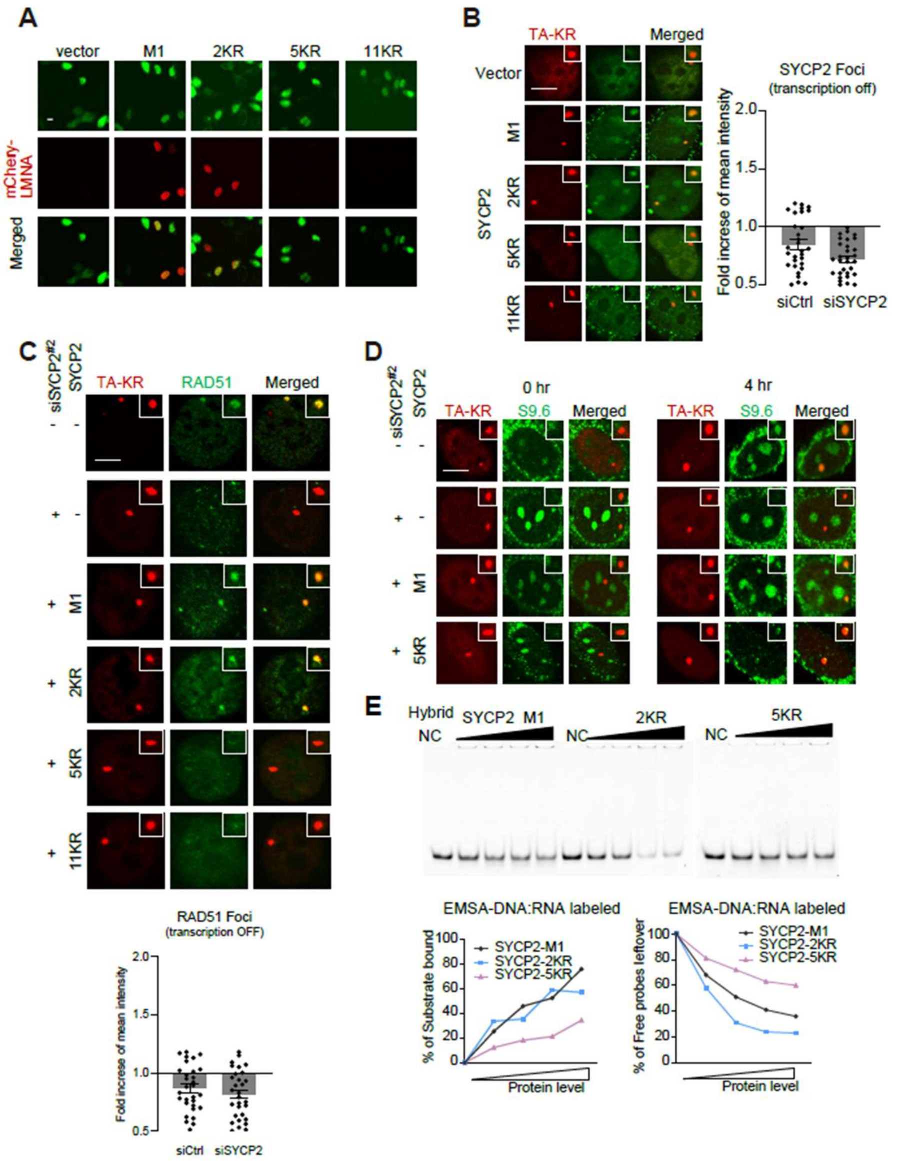

Supplementary Figure 5. Lysine (K)/Arginine(R) motif in the SYCP2 M1 domain is required for R-loop stabilization and TC-HR. A. Representative images of CRISPR-based LMNA reporter assay in U2OS reporter cells with each indicated M1 mutants' expression. **B.** Left: Representative images of SYCP2 M1 or its mutant'

localization at TA-KR sites in siSYCP2#2 pretreated U2OS cells. Right: Fold increase of endogenous SYCP2 foci at sites of tetR-KR compared to background was quantified (n=30, mean+/- SD). **C.** Upper: Representative images of RAD51 localization at TAKR sites in siSYCP2#2 pretreated U2OS cells with expression of M1 or indicated M1 mutants. Lower: Fold increase of endogenous RAD51 foci at sites of tetR-KR compared to background was quantified (n=30, mean+/- SD). **D.** Representative images of S9.6 staining in siSYCP2#2 pretreated U2OS-TRE cells with expression of SYCP2-M1 or -5KR mutant or empty vector. **E.** *In vitro* binding of purified SYCP2-M1 and its mutant protein with DNA: RNA hybrids (0.1μM) were analyzed in EMSA. Statistical analysis was done with the Student-t-test. Scale bar = 10 μm.

Supplementary Figure 6

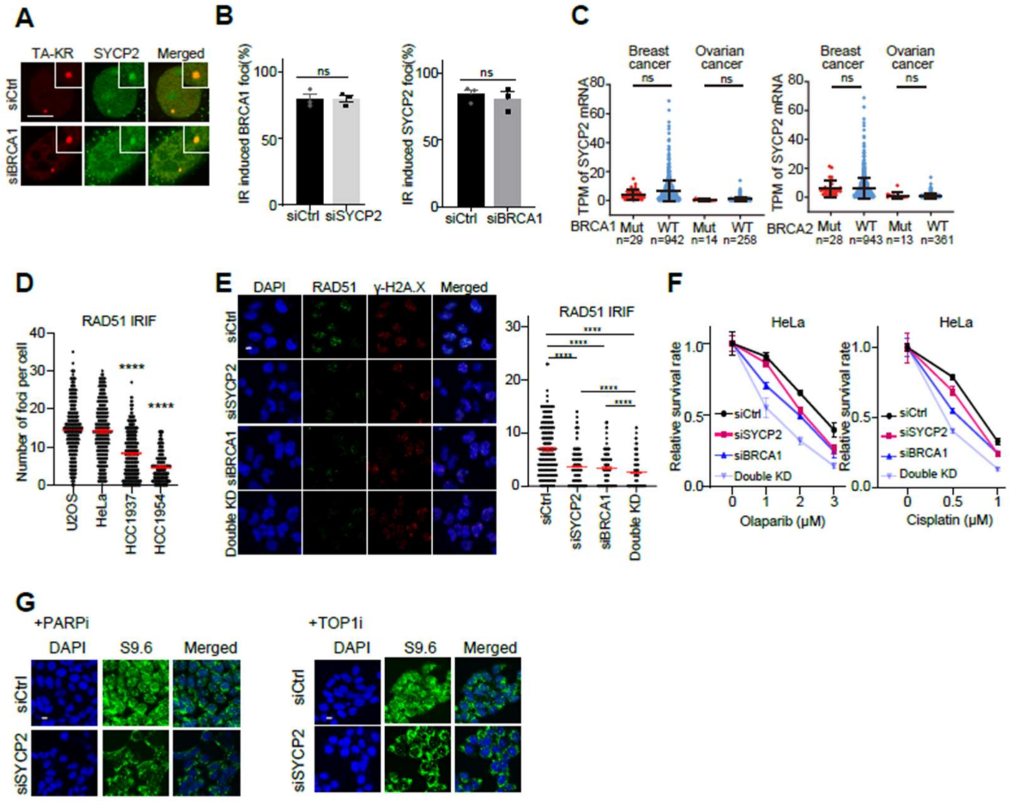

Supplementary Figure 6. Function of SYCP2 is independent of BRCA1. A. Representative images of SYCP2 at TA-KR sites in siSYCP2 knockdown U2OS cells. **B.** IRIF of BRCA1 and SYCP2 colocalizing with γH2AX in U2OS cells 1 hr after 2 Gy IR were quantified in U2OS cells (n=3, mean+/- SD). **C.** Comparison of SYCP2 RNA expression from CCLE database in BRCA1/2 mutant or proficient breast cancer cell lines. The analysis was normalized to Transcripts Per Million (TPM). **D.** The numbers of RAD51 IRIF in U2OS, HCC1937 and HCC1954 after 2 Gy IR after 1 hr recovery. Three experiments were done (n=200, mean+/- SD). **E.** The numbers of RAD51 IRIF in siCtrl, siBRCA1, siSYCP2, or siBRCA1+siSYCP2 treated U2OS cells 1 hr after 2 Gy IR were quantified (n=200, +/- SD). The representative images of RAD51 in siCtrl, siBRCA1, siSYCP2, or siBRCA1+siSYCP2 treated U2OS were shown on the left. **F.** Cell survival rate of HeLa cells with siCtrl, siBRCA1, siSYCP2, or

siBRCA1+siSYCP2 via colony-forming assay with the treatment of Olaparib or Cisplatin at indicated dose. **G.** Staining of S9.6 in U2OS cells before or after treatment of 1 μM Olaparib and 1 μM CPT11 for 24 hrs. Statistical analysis was done with the Student-t-test, ****: $p<0.0001$. Scale bar = 10 μm.

Supplementary Figure 7

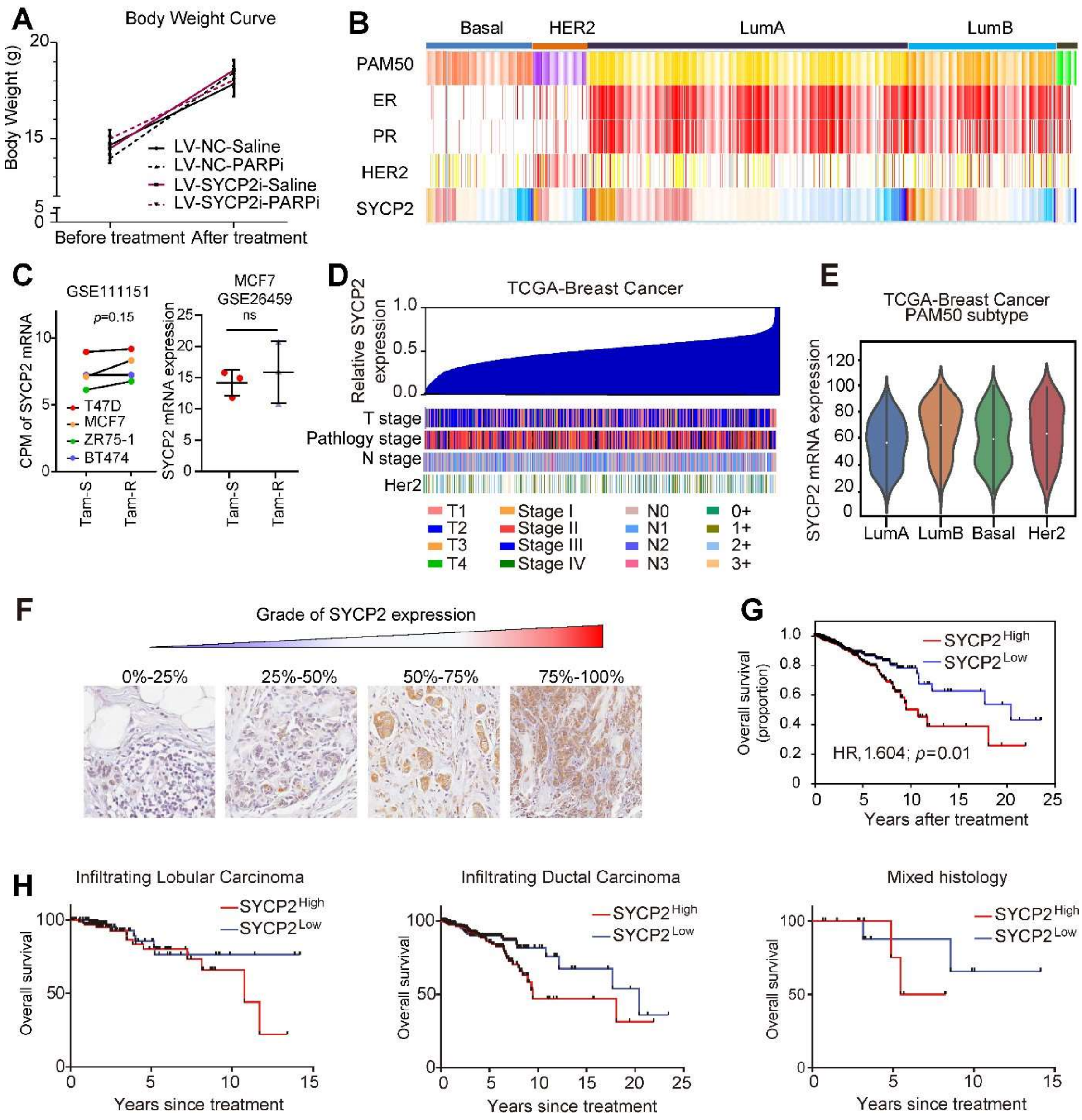

Supplementary Figure 7. SYCP2 expression is a biomarker of poor prognosis in patients. A. The average body weight of mice with indicated lentiviruses (LV) treatment before and after Olaparib or Saline treatment. **B.** The heatmap of correlation between PAM50, ER, PR, HER2 and SYCP2 levels and different types of breast cancer (Basal, HER2, LumA and LumB). **C.** Left: The comparison of SYCP2 expression levels of breast cancer cell lines (T47D, MCF7, ZR75-1 and BT474) between the group of Tamoxifen sensitive versus Tamoxifen resistant according to the GDSC database (GSE111151). Right: The comparison of SYCP2 expression in

Tamoxifen sensitive versus Tamoxifen resistant in MCF7 cell lines (GSE26459). **D.** Expression of SYCP2 in breast cancer samples from TCGA database (n=1217). The corresponding status of Tumor stages, pathology stages, lymph node stage, and Her2 levels are indicated as the labeled colors. **E.** Violin plot of SYCP2 expression in PAM50 subtypes of breast cancer. (LumA n=618, LumB n=156, TNBC n=62, Normal n=113.) **F.** Representative images of the guideline for levels of SYCP2 in classifying patients' group. Patients are divided by the positive staining at indicated ranges. **G.** Overall survival curves of 1095 patients from TCGA. Cutoff value is median. R-value and p-value were calculated by COX survival analysis. **H.** Overall survival curves of different histology types of breast malignant tumors (infiltrating lobular carcinoma (ILC), infiltrating ductal carcinoma (IDC) and mixed histology) from analyzing data from TCGA. Cutoff value is median. R value and p value were calculated by COX survival analysis.

Supplementary Figure 8

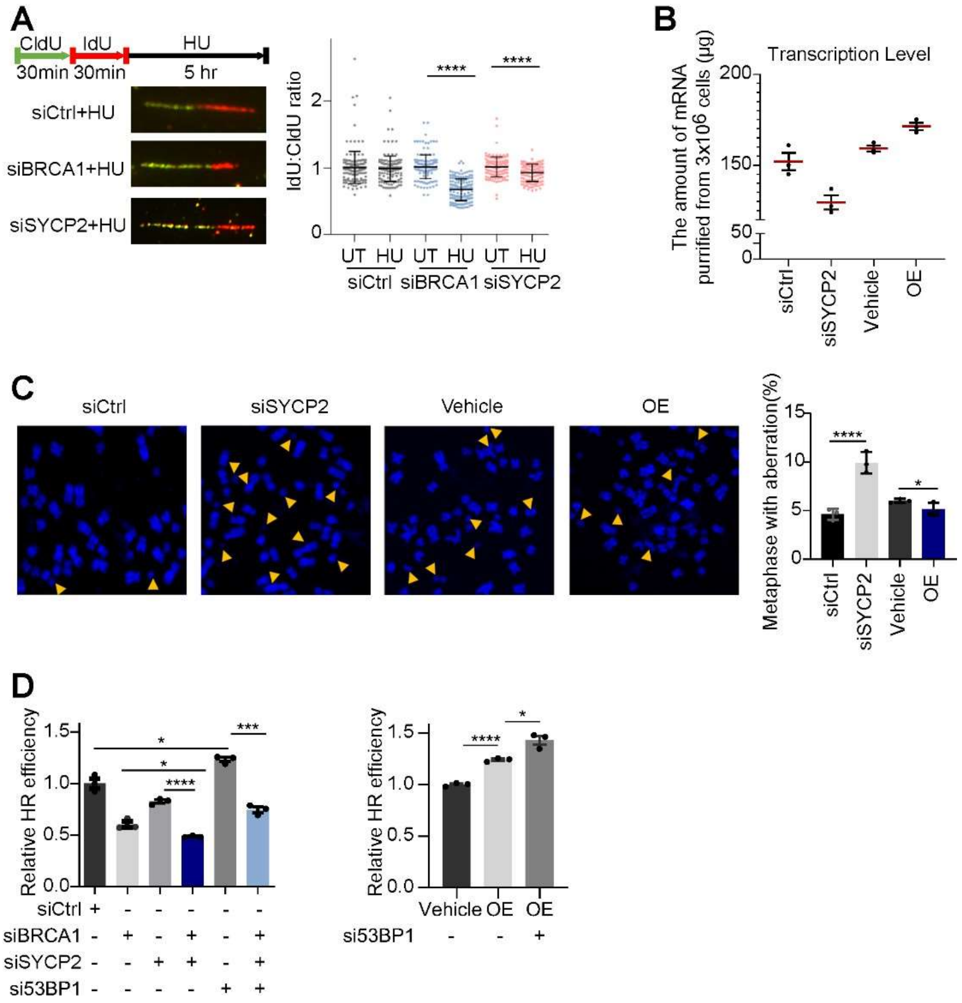

Supplementary Figure 8. Function of SYCP2 in other cellular processes. A. DNA fiber assay was done in U2OS cells were treated with siBRCA1 or siSYCP2 with or without [untreated (UT)] 4 mM HU for 5 hours. The length of labeled CldU/IdU tracks was measured via Fiji software, and ratios of IdU and CldU length (each group, $n > 660$) are shown. **B.** Quantification of mRNA purified from siCtrl or siSYCP2 or empty vector or SYCP2 OE treated U2OS cells. **C.** representative images of metaphases of U2OS cells treated with siSYCP2 or siCtrl or empty vector or SYCP2 OE. Quantification of incidence of total chromosomal aberrations ($n > 500$ in one experiment). Three independent experiments were done (n=3, +/- SD). **D.** Relative HR frequency (FACs) in siCtrl or siBRCA1 or siSYCP2 or siBRCA1+siSYCP2 or si53BP1 or siBRCA1+siSYCP2+si53BP1 (left) or empty vector or SYCP2 overexpression (OE) or SYCP2-OE+si53BP1 (middle) or siCtrl or siBRCA1 or siSYCP2

or siBRCA1+siSYCP2 (right) in the DR-GFP reporter assay. the total amount of mRNA purified from three million cells of siCtrl or siSYCP2 or empty vector or SYCP2 OE treatment. Statistical analysis was done with the Student-t-test, **P* < 0.05, ***: p<0.001, ****: p<0.0001.

Supplementary Table 1

**Supplementary Table 1**	
**Abbreviations**	**Full name**
ACC	Adrenocortical carcinoma
BLCA	Bladder Urothelial Carcinoma
BRCA	Breast invasive carcinoma
CESC	Cervical squamous cell carcinoma and endocervical adenocarcinoma
CHOL	Cholangiocarcinoma
COAD	Colon adenocarcinoma
DLBC	Lymphoid Neoplasm Diffuse Large B-cell Lymphoma
ESCA	Esophageal carcinoma
GBM	Glioblastoma multiforme
HNSC	Head and Neck squamous cell carcinoma
KICH	Kidney Chromophobe
KIRC	Kidney renal clear cell carcinoma
KIRP	Kidney renal papillary cell carcinoma
LAML	Acute Myeloid Leukemia
LGG	Brain Lower Grade Glioma
LIHC	Liver hepatocellular carcinoma
LUAD	Lung adenocarcinoma
LUSC	Lung squamous cell carcinoma
OV	Ovarian serous cystadenocarcinoma
PAAD	Pancreatic adenocarcinoma
PCPG	Pheochromocytoma and Paraganglioma
PRAD	Prostate adenocarcinoma
READ	Rectum adenocarcinoma
SARC	Sarcoma
SKCM	Skin Cutaneous Melanoma
STAD	Stomach adenocarcinoma
TGCT	Testicular Germ Cell Tumors
THCA	Thyroid carcinoma
THYM	Thymoma
UCEC	Uterine Corpus Endometrial Carcinoma
UCS	Uterine Carcinosarcoma

The full name of all types of cancer used in the analysis is listed in **Table S1.**

Supplementary Materials and Methods

Metaphase chromosome spreading assay

U2OS cells were grown on 6 well plates 24 hours before transfection of siRNA and plasmid transfection. For analyzing the chromosomal aberration, siRNA targeting SYCP2 or empty plasmid vector or SYCP2 overexpression plasmid were transfected into the cells for over 36 hours expression. 100 μg/ml Colcemid (10 μ g/ml; Gibco BRL) was added to the cells, then cells were incubated at 37°C for 3 hours. Cells were gently removed of culture media and added 10ml of PBS with gentle pipetting. The floating cells were collected and centrifuged. Left 2ml of PBS and cell pellet, added 8 ml of 75 mM KCl and gently mixed and incubated at 37°C for 30 min. Cells were fixed using the freshly made methanol:glacial acetic acid solution as a ratio of 3:1 three times. To made the slides, applied three or four drops of cell suspension to the microscope slides set at a 45° vertical angle and washed with fixative solution and leave for drying. The dried slides were stained with DAPI (Sigma-Aldrich) and mounted with DABCO (1,4-diazabicyclo[2.2.2]octane).

RNA extraction, cDNA synthesis, and quantitative real-time polymerase chain reaction (RT-PCR)

Frozen tumor tissues (including 5 tumor and one normal adjacent samples) were minced using a pestle and mortar into small pieces and total RNA was extracted using the Purelink RNA minikit (Invitrogen). Cells were collected and centrifuged for 5 min at 500 x g and washed in phosphate-buffered saline twice (PBS; Gibco). Total RNA was also isolated from cell pellets using the Purelink RNA minikit (Invitrogen). mRNA was isolated using the Dynabeads™ mRNA Purification Kit from Invitrogen. cDNA was obtained through reverse transcription of 500ng purified RNA via Quantinova Reverse Transcription kit (Qiagene). cDNA was amplified using primers (TTGGAAAAGGGACAGCCAAG) and (GGTTGCTTTTCGTGGAAGTCTG) targeting SYCP2, primers (TTCACCACCATGGAGAAGGC) and (TCTCATGGTTCACACCCATGAC) targeting GAPDH and primers (AGAGCTACGAGCTGCCTGAC) and (AGCACTGTGTTGGCGTACAG). Each reaction was labeled via the QuantiNova SYBR Green PCR Kit (Qiagene) and performed using StepOnePlus™ Real-Time PCR System (Applied Biosystems). Reactions of each sample were triplicated in an individual experiment; the final results

came from three individual experiments. C_T value of normal tissue was set as control for expression calculation. The expression levels were normalized based on the housekeeping gene *GAPDH*.

Cell cycle analysis

The cells were collected and fixed in cold 70% ethanol at 4 °C overnight. The cells were washed once with 2% BSA in PBS and incubated in PBS solution containing 2% BSA, 50 μg/mL propidium iodide, and 100 μg/mL RNase A in the dark for 30 min before being analyzed by flow cytometry.

Repair reporter Assays

HR assays using DR-GFP were described in the previous study [1]. Cells were seeded into 6-well plates and transfected with pCMV-I-SceI plasmid. The normal cell population was gated in PI by SSC-A and FSC-A. Each flow cytometry experiment was conducted with over 50,000 cells. The HR rate was then calculated from the population of GFP-positive cells. EJ5, EJ2, SSA reporter assays were conducted following the procedure described in Yang et al[2].

Protein purification

The DNA fragment encoding SYCP2-M1 (492-1035 a.a.) was cloned into pENTER/D-TOPO (Invitrogen). The M1 fragment then transferred to pDEST17 gateway vector for the protein expression using T7 Express *lysY/I^q* Competent *E. coli* (NEB, C3013) as a host strain. For the protein purification, the *E. coli* cells harboring PDEST17-M1 that encode the functional domain of SYCP2 with a N-terminal 6X His tag was grown to an optical density of 0.4 and then induced by the supplement of 0.3 mM Isopropyl β-D-1-thiogalactopyranoside (IPTG) for 16 h at 16°C. The overexpressed cells were lysed in buffer (25 mM Tris-HCl, pH 8.0), 300mM NaCl, 1 mM EDTA, 10 % glycerol, 1 mM DTT, 1 mM PMSF, protease inhibitors) and sonicated. The lysed sample was centrifuged for 1 h at 12,000 r.p.m followed by passing through a 0.45 μm filter to remove the cell debris. The cleared supernatant was incubated with Ni-NTA agarose beads overnight and then washed with washing buffer (80mM imidazole, 25 mM Tris-HCl, pH 8.0), 300mM NaCl). The protein was finally eluted by buffer containing 200mM imidazole, 25 mM Tris-HCl, pH 8.0, and 300mM NaCl followed by concentration with a 30 kDa cutoff concentrator. The imidazole was also removed in the concentration process. The final purified protein was aliquoted and stored at − 80 °C freezer for the biochemical assays.

DNA End Resection Assay

Resection assay was performed using a similar procedure as described in Zhou. et. al., 2014[3]. Differences from the previous procedure are described as follows. The ER-*Asi*SI U2OS cells were grown in DMEM medium supplemented with 10% FBS (Atlas Biologicals, Fetal Select) and 1X Pen/Strep/Glutamine (Thermo Fisher) at 37^0C with 5% CO_2. The cells were grown to 60%-70% confluence on a 3.5cm dish prior to each transfection. The cells were transfected with *siControl*, *siSYCP2* or *siMRE* using lipofectamine RNAimax transfection reagent according to Invitrogen for 24 hours using the manufacture recommended conditions. After transfection, siRNAs were washed out with warm medium, and 300nM 4-hydroxytamoxifen (4-OHT) (Sigma) was used to induce double strand breaks (DSBs) for 4 hours. After DNA extraction, 100ng of total DNA was digested with either BsrGI-HF or BamHI-HF for assessing resection loci for DSB1 or DSB2 respectively. TB Green Premix Ex Taq (Takara) or Power SYBR Green PCR Master Mix (Applied Biosystems) was used to perform quantitative PCR (qPCR) on a LightCycler 480 II (Roche) or QuantStudio (Applied Biosystems) qPCR machine. Primer information and the quantification of percentage of ssDNA are described in previous methods[3].

DNA Fiber assay

Cells were seeded in 6-cm dish and transfected with siCtrl and siBRCA1/siSYCP2. After 24-36 hours transfection, cells were labeled with 25μM CldU (Sigma-Aldrich, C6891mg) for 30 min. Cells were then washed with PBS twice and labeled with 250Mm IdU (Sigma-Aldrich, I7125-5g) for another 30 min. Then cells were treated with or without 4mM HU for 5 hours. DNA fibers were prepared onto slides according to the protocol from Buisson et al. (PMID: 26365377). The slides were incubated with primary antibodies anti-CldU [rat monoclonal anti–5-bromo-2′-deoxyuridine (BrdU)/CldU; BU1/75 ICR1, Novus, 1:100] and anti-IdU (mouse monoclonal anti-BrdU/IdU; clone B44, Becton Dickinson, 1:50) covered with glass coverslip in a humidified chamber for 30 min at 37°C. And secondary antibodies goat anti-mouse Alexa Fluor 594 (Abcam, ab150116, 1:200) or goat anti-rat Alexa Fluor 488 (Abcam, ab150157, 1:200) were used for incubation for 1 hour at room temperature. Images were taken under a Nikon Eclipse 80i fluorescence microscope from fields of untangled fibers. The length of labeled CldU/IdU tracks was measured via Fiji software, the calculation was done by dividing the length of CldU

over IdU, and the mean of at least three independent experiments is presented. Statistical analysis was performed using GraphPad Prism Software.

Supplementary References:

1. Chen, H. *et al.* m(5)C modification of mRNA serves a DNA damage code to promote homologous recombination. *Nat Commun* **11**, 2834 (2020).
2. Yang, H. *et al.* The RNA m5C modification in R-loops as an off switch of Alt-NHEJ. *Nat Commun* **14**, 6114 (2023).
3. Zhou, Y., Caron, P., Legube, G. & Paull, T.T. Quantitation of DNA double-strand break resection intermediates in human cells. *Nucleic Acids Res* **42**, e19 (2014).
