# Supplementary material for: Meiotic protein SYCP2 confers resistance to DNA-damaging agents through R-loop-mediated DNA repair
Source: Nat Commun. 2024 Feb 21;15:1568. doi: 10.1038/s41467-024-45693-2 (PMC10881575; doi:10.1038/s41467-024-45693-2)
Supplement: Supplementary file 3 — Reporting Summary [file 41467_2024_45693_MOESM3_ESM.pdf]

nature portfolio

Corresponding author(s): Li Lan

Last updated by author(s): 1/5/2024

Reporting Summary

Nature Portfolio wishes to improve the reproducibility of the work that we publish. This form provides structure for consistency and transparency in reporting. For further information on Nature Portfolio policies, see our Editorial Policies and the Editorial Policy Checklist.

Statistics

For all statistical analyses, confirm that the following items are present in the figure legend, table legend, main text, or Methods section.

n/a	Confirmed	
☐	☒	The exact sample size (n) for each experimental group/condition, given as a discrete number and unit of measurement
☐	☒	A statement on whether measurements were taken from distinct samples or whether the same sample was measured repeatedly
☐	☒	The statistical test(s) used AND whether they are one- or two-sided<br>*Only common tests should be described solely by name; describe more complex techniques in the Methods section.*
☒	☐	A description of all covariates tested
☒	☐	A description of any assumptions or corrections, such as tests of normality and adjustment for multiple comparisons
☐	☒	A full description of the statistical parameters including central tendency (e.g. means) or other basic estimates (e.g. regression coefficient) AND variation (e.g. standard deviation) or associated estimates of uncertainty (e.g. confidence intervals)
☐	☒	For null hypothesis testing, the test statistic (e.g. F, t, r) with confidence intervals, effect sizes, degrees of freedom and P value noted<br>*Give P values as exact values whenever suitable.*
☒	☐	For Bayesian analysis, information on the choice of priors and Markov chain Monte Carlo settings
☒	☐	For hierarchical and complex designs, identification of the appropriate level for tests and full reporting of outcomes
☐	☒	Estimates of effect sizes (e.g. Cohen's d, Pearson's r), indicating how they were calculated

Our web collection on statistics for biologists contains articles on many of the points above.

Software and code

Policy information about availability of computer code

Data collection	FV1000 confocal software 4.2, Step one V2.3, ChemiDocTM MP imaging system
Data analysis	ImageJ 1.51k, Graphpad prism 7.01, Flowjo 10.6.2

For manuscripts utilizing custom algorithms or software that are central to the research but not yet described in published literature, software must be made available to editors and reviewers. We strongly encourage code deposition in a community repository (e.g. GitHub). See the Nature Portfolio guidelines for submitting code & software for further information.

Data

Policy information about availability of data

All manuscripts must include a data availability statement. This statement should provide the following information, where applicable:

- Accession codes, unique identifiers, or web links for publicly available datasets
- A description of any restrictions on data availability
- For clinical datasets or third party data, please ensure that the statement adheres to our policy

The main data supporting the results of this study are available within the Article and its supplementary information. Public RNA sequencing (RNAseq) data for cell lines were obtained from the CCLE (Cancer Cell Line Encyclopedia) project (https://portals.broadinstitute.org/ccle) and GDSC (Genomics of Drug Sensitivity in Cancer) project (https://www.cancerrxgene.org/). All expression data were processed to TPM by using Python. Expression data from CCLE was normalized by using ln (TPM+1). RNAseq data of the gene expression are available for download at TCGA (https://www.cancer.gov/), GTeX(https://gtexportal.org/), TARGET(https://

software.broadinstitute.org/cancer/cga/target), and treehouse (https://treehousegenomics.soe.ucsc.edu/public-data/). The full name of all types of cancer used in the analysis is shown in Supplementary Table 1.

Human research participants

Policy information about studies involving human research participants and Sex and Gender in Research.

Reporting on sex and gender	The occurrence of breast and ovarian cancer is almost exclusive in woman. All patients in this study were female, and their gender was not considered in this study.
Population characteristics	Famale breast cancer patients, ovarian cancer patients, who had been diagnosed and had cancer treatments. The confounding factors in this study includes tumor status, lymph nodes status, pathological status and HER2 levels. Female or male patients ≥ 18 years of age. The median age = 48.5.
Recruitment	16 breast cancer patients from the Massachusetts General Hospital Cancer Center, 28 ovarian cancer patients who provided informed consent to an Institutional Review Board approved banking trial. There are no potential biases in selecting test candidates.
Ethics oversight	The breast tissue samples were collected under the approval of Institutional Review Board. The coded ovarian tumor samples along with patients' deidentified clinical data were obtained under a secondary use Institutional Review Board approved protocol by Massachusetts General Hospital(#2014P002048).

Note that full information on the approval of the study protocol must also be provided in the manuscript.

Field-specific reporting

Please select the one below that is the best fit for your research. If you are not sure, read the appropriate sections before making your selection.

☒ Life sciences ☐ Behavioural & social sciences ☐ Ecological, evolutionary & environmental sciences

For a reference copy of the document with all sections, see nature.com/documents/nr-reporting-summary-flat.pdf

Life sciences study design

All studies must disclose on these points even when the disclosure is negative.

Sample size	Sample size, number of replicates, error bars and statistical tests were chosen based on accepted practices in the field and stated in each figure legend. Generally, experiments were performed independently and reproduced using at least three biological replicates. over 10 cells per condition and biological replicate in a group were analyzed per intensity purpose. At least 50 cells per condition and biological replicate were counted for frequency in IF experiments. 200 cells were counted for IRIF experiments.
Data exclusions	No data was excluded in our analysis.
Replication	Each experiment was repeated 2-3 times with similar results.
Randomization	Each animal were chosen randomly. When individual cells were analyzed in cell populations, they were randomly selected from the populations.
Blinding	The investigators were not blinded during data collection. Blinding is not relevant for the study, results were calculated, counted or analyzed by software, or different performers for unbiased purpose.

Reporting for specific materials, systems and methods

We require information from authors about some types of materials, experimental systems and methods used in many studies. Here, indicate whether each material, system or method listed is relevant to your study. If you are not sure if a list item applies to your research, read the appropriate section before selecting a response.

Materials & experimental systems

n/a	Involved in the study	
☐	☒	Antibodies
☐	☒	Eukaryotic cell lines
☒	☐	Palaeontology and archaeology
☐	☒	Animals and other organisms
☒	☐	Clinical data
☒	☐	Dual use research of concern

Methods

n/a	Involved in the study	
☒	☐	ChIP-seq
☐	☒	Flow cytometry
☒	☐	MRI-based neuroimaging

Antibodies

Antibodies used

Antibody Species Clone, Catalog no. Company
SYCP2 Rabbit polyclonal, LS-C386874 LifeSpan BioSciences
SYCP2 Rabbit polyclonal, PA5-67554 Invitrogen
β-actin Mouse monoclonal (8H10D10) , #3700 Cell Signaling Technology
RAD51 Rabbit polyclonal, Ab63801 Abcam
BRCA1 Mouse monoclonal (D-9), sc-6954 Santa Cruz Biotechnology
Cyclin A Mouse monoclonal (B-8), sc27162 Santa Cruz Biotechnology
CtIP Mouse monoclonal (clone14-1) #61142 Active Motif
RPA Rabbit polyclonal (RPA70/1) #2267 Cell Signaling Technology
pRPA Rabbit polyclonal A300-246A Bethyl
S9.6 Mouse monoclonal, ENH001 Kerafast
GFP Mouse monoclonal, 11814460001 Roche
Ki-67 Mouse monoclonal, sc-23900 Santa Cruz Biotechnology
ɣH2AX, ser139 Mouse monoclonal, JBW301, 05–636 EMD Millipore

Validation

All antibodies are commercially available and have been validated by suppliers and previous publications. All validations could be obtained by the websites of each company. Antibodies were used according to manufacture instructions

Eukaryotic cell lines

Policy information about cell lines and Sex and Gender in Research

Cell line source(s)

HCC1954, HCC1937, MDA-MB-231, MDA-MB-468, MCF10A, MCF7, T47D, BJ, HeLa, U2OS TRE cell

Authentication

Cell lines were not authenticated. HCC1954, HCC1937, MDA-MB-231, MDA-MB-468, MCF10A, MCF7, T47D, BJ, HeLa and U2OS cells were purchased from ATCC and integrated with TRE repeats.

Mycoplasma contamination

No Mycoplasma contamination of cell lines. BM-cyclin (Sigma-Aldrich Cat 1079905001) was used during culture and removed before experiments.

Commonly misidentified lines
(See ICLAC register)

no commonly misidentified line were used

Animals and other research organisms

Policy information about studies involving animals; ARRIVE guidelines recommended for reporting animal research, and Sex and Gender in Research

Laboratory animals

Species and Strain: BALB/c nude mice, Weight:20g, Age:4-5 weeks

Wild animals

no wild animals were used

Reporting on sex

Sex: Female,

Field-collected samples

no filed-collected samples

Ethics oversight

All animal experiments were approved by and conducted in accordance with the guidelines established by the Institutional Animal Care and Use Committee at the Massachusetts General Hospital with the protocol number 2003N000186.

Note that full information on the approval of the study protocol must also be provided in the manuscript.

Flow Cytometry

Plots

Confirm that:

☒ The axis labels state the marker and fluorochrome used (e.g. CD4-FITC).

☒ The axis scales are clearly visible. Include numbers along axes only for bottom left plot of group (a 'group' is an analysis of identical markers).

☒ All plots are contour plots with outliers or pseudocolor plots.

☒ A numerical value for number of cells or percentage (with statistics) is provided.

Methodology

Sample preparation	the sample preparation has been described in Materials and Methods section
Instrument	LSR II
Software	flowjo 10.6.2
Cell population abundance	at least 10,000 cells were collected for each experiment
Gating strategy	The normal cell population was gated in P1 by SSC-A and FSC-A. The HR rate was then calculated as the ratio of GFP-positive cells number to mCherry –positive cells number.

☒ Tick this box to confirm that a figure exemplifying the gating strategy is provided in the Supplementary Information.
